# Study protocol - resilience in individuals and families coping with the impacts of alcohol related injuries in remote indigenous communities: a mixed method study

**DOI:** 10.1186/1471-2458-14-479

**Published:** 2014-05-21

**Authors:** Caryn West, Kim Usher, Alan R Clough

**Affiliations:** 1 School of Nursing, Midwifery & Nutrition, James Cook University, PO Box 6811, Cairns, QLD 4870, Australia; 2 School of Health, University of New England, Armidale, NSW 2531, Australia; 3 Community-based Health Promotion and Prevention Studies Group, Australian Institute of Tropical Health and Medicine, James Cook University (Cairns Campus), PO Box 6811, Cairns, Qld 4870, Australia

**Keywords:** Alcohol, Injury, Resilience, Indigenous health

## Abstract

**Background:**

Alcohol Management Plans (AMPs) were first implemented by the Queensland Government a decade ago (2002–03). In 2008, further stringent controls were implemented and alcohol was effectively prohibited in some of the affected remote Indigenous communities. With the Queensland Government currently reviewing AMPs, prohibitions may be lifted making alcohol readily available once more in these communities. As yet no work explores the impact of alcohol related injuries in relation to individual, family and community resilience in Indigenous Australians. A resilience model recognises individuals and families for their strengths rather than their deficits. By revealing how some individuals and families survive and thrive, new ways of working with families who need support may be identified and adopted. The research will explore in detail the long-term impact of this kind of injury on individuals, families and communities.

**Methods/design:**

This project will use a sequential explanatory mixed method design. Four discrete Indigenous communities in Cape York, far north Queensland are included in this program of research, chosen because there is previous data available regarding injury and alcohol related injuries. Four sequential studies will be conducted in order to address the research questions and provide a rich description of the impact of alcohol related injuries and resilience in these populations. The time period January 2006 to December 2011 was chosen because it captures the three years before and three years after 2008 when tight alcohol restrictions were implemented in the four communities.

**Discussion:**

Long term effects of the AMPs are as yet unknown and only fragmented attempts to look at the impact of injury related to alcohol have been conducted. A well-structured research program that explores the long-term impact of alcohol related injuries in these communities will help inform policy development to capture the current situation and so that appropriate benchmarking can occur.

The project has been approved by the James Cook University Human Research Ethics Committee H5618 & H5241.

## Background

This project aims to describe and categorise injuries in four remote Indigenous communities in Cape York, far north Queensland, Australia. In the four Indigenous Australian communities included in this research program, and in others like them across Queensland, Alcohol Management Plans (AMPs) were first implemented by the Queensland Government just over a decade ago (2002–03) [1,2]. The AMPs were initially focused on controlling alcohol availability, mainly by prohibiting or limiting the alcohol taken into these communities, known as ‘carriage limits’. More recently, at the end of 2008, in three of the study communities, alcohol became locally prohibited with closure of community ‘canteens’ where these alcohol outlets were operating, and it was further restricted in the fourth community with its canteen becoming a community-run ‘sports and social club’. Closely linked to Indigenous health outcomes the AMPs aimed at reducing alcohol related violence and injury. However, there remains a paucity of rigorous data to fully describe the positive changes in injury rates linked with Queensland’s controversial AMPs [3,4]. The limited available published evidence is supported by anecdote and it indicates that there have been some successes due to the AMPs, possibly changing a long term pattern of alcohol misuse which has had devastating effects in many remote Indigenous communities in Queensland [4,5]. The current Queensland Government has AMPs under review, bringing the prospect that alcohol may become readily available once more.

Initially the AMPs (2002–03) seemed to have had a number of positive impacts on injury rates, however the results varied across communities [5]. Data for the period 1995–2005 described Royal Flying Doctor Service (RFDS) aero-medical retrievals for serious injury in the four study communities which indicates substantial reductions in this indicator of serious injury [3]. However, two years after the 2002–03 AMP implementations, aero-medical retrievals again began to rise [3]. RFDS data on aero-medical retrievals for serious injury was again reviewed for the period 1996–2010 [5] showing overall, serious injury retrieval rates decreased significantly to what were, at that time, the lowest levels seen in 15 years [5]. Whether these interesting trends in injury indicators have declined in other Indigenous communities affected by AMPs is an important unanswered question. However, for this study, the focus is on all injury, particularly on whether these positive results for serious injury mean that all injuries in the study communities have decreased.

Only one study has ever tried to describe injuries in Cape York in any detail [4] and no studies have examined the long-term consequences of the injuries suffered and their impact on individuals and families. A 1997 study of injury in five Cape York communities (including the four in the proposed research program) found that alcohol related injuries accounted for 50.7% of all injury sustained, with men (53%) and women (47%) similarly likely to sustain an alcohol related injury [4]. These findings are now almost 20 years old and although useful as a reference point, and the only in-depth evidence describing injuries, they may not accurately represent the current situation or reflect any of the more recent effects that alcohol prohibition and control may have had on injuries sustained within the communities.

As with most adversity it is not solely the individual who bears the impact of the event [6] When injury and illness occur; individuals, families, communities and healthcare systems are also impacted. As yet, there has been no work which explores the impact of alcohol related injuries in relation to individual, family and community resilience in Indigenous Australians. Researchers and healthcare providers advocate a shift from problem-based solutions to strength-based solutions [6-8]. The significance of a resilience model for such a shift in emphasis is that it recognises individuals and families for their strengths. Importantly, resilience does not ignore the problems present or treats them as deficits; but rather focuses on how people cope with them. What is of particular importance for potential interventions is that resilience can be learnt [8]. By revealing how some individuals and families survive and thrive, new ways of working with families who need support may be identified and adopted [6].

## Aim

With a focus on alcohol related injuries in four Indigenous communities in Cape York, Queensland, the research will explore in detail the long-term impact of this kind of injury on individuals, families and communities. The goal is to better understand resilience strategies that may have helped to minimise alcohol’s negative effects. A sequential explanatory mixed method design will be used in the program to address the following research questions:

1. **What have been the numbers and types of injuries suffered by community people and have these changed in recent years?** - A clinical file audit will be performed in the four communities to describe injuries occurring during the period January 2006 through December 2011. Data for this time period is included because it will permit a comparison between the three years before and three years after local prohibition and tighter controls on alcohol were enforced in the four communities at the end of 2008.

2. **What has been the role of alcohol in these injuries during these years? –** In the clinical file audit, alcohol related injuries will be identified for further exploration.

3. **What impacts have alcohol related injuries had on individuals and families? –**The Impact of Event Scale-Revised (IES-R) will be used to assess trauma resulting from alcohol related injury. Where identified, this will be explored in depth using qualitative interviews.

4. **What is the role of resilience for individuals and families when a family member has suffered an alcohol related injury? –** The Connor-Davidson Resilience Scale (CD-RISC) will be used to assess levels of resilience. The degree and type of resilience will also be explored using in depth interviews.

5. **What strategies can enhance resilience to minimise any future negative effects of alcohol related injuries? –** A Delphi study will be performed in each community to prioritise resilience strategies that arise from the interviews.

## Methods/design

### Setting & location

**Figure 1:**
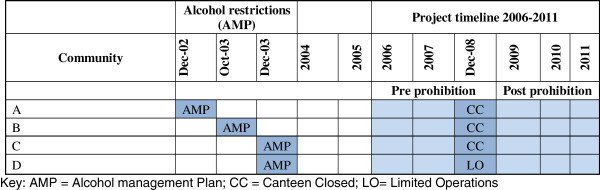
**Introduction of alcohol restrictions in four study communities in Cape York.** Key: AMP = Alcohol management Plan; CC = Canteen closed; LO = Limited operations.

Four discrete Indigenous communities in Cape York are included in this program of research. These communities were chosen as there is previous data available from two studies which mapped injury rates and aero-medical retrievals [3,4] and from a clinical audit conducted in the mid-1990s. Controls on the availability of alcohol were implemented in these four communities as outlined in Figure 1[9].

Current data indicates a total study population of n = ~ 3694 across the four communities (Table 1) [10].

**Table 1:** Populations in four Cape York communities

**Community**	**Pre prohibition population: 2006 ABS census data (10)**	**Post Prohibition population: 2011 ABS census data (10)**
A	1043	1296
B	542	483
C	1017	1207
D	600	708

### Participants and recruitment

Inclusion criteria are: i) adult (18 years and older); ii) Indigenous Australian, i.e. Aboriginal or Torres Strait Islander or both; iii) sustained an injury during the period January 2006 to December 2011; iv) sustained an alcohol related injury during the period January 2006 to December 2011, or be a family member of an individual who has sustained an alcohol related injury during the period January 2006 to December 2011. This time period was chosen because it captures the three years before and three years after 2008, when more stringent alcohol controls were implemented in the four communities.

### Funding

Funding for the study was provided by the National Health and Medical Research Council of Australia (NHMRC, Early Career Fellowship #1070931).

### Ethics

The study was approved by the Human Research Ethics Committee James Cook University (H5618 & H5241). As part of a larger evaluation project (NHMRC project grant #APP1042532) [11], this project was considered and supported by the Indigenous Leaders’ Forum of the Local Government Association of Queensland which included the duly elected Mayors and CEOs from all affected Queensland communities. Approval was also sought from the following Human Research Ethics Committees: Cairns and Hinterland Health Services District Human Research Ethics Committee (HREC/14/QCH/3 – 883) and Townsville and District Human Research Ethics Committee (HREC/13/QTHS/187).

### Consent

Permission for consent to be waivered for the clinical file audit has been granted by the appropriate Health Services District Human Research Ethics Committees. For remaining project elements individuals indicating that they wish to participate will be provided with written information about the study and asked to sign a consent form. Participants will be asked to provide separate consent for various elements of the project including the use of audio recording devices. All participants will be informed that their participation is voluntary and that they can refuse or withdraw from the study without reason or justification for their decision. The processes for seeking consent to participate meet the NHMRC Guidelines for Ethical Conduct in Aboriginal and Torres Strait Islander Health Research [12].

## Study overview and choice of study design

A sequential explanatory mixed method design will be used for this project. The research program is comprised of four sequential studies in order to address the research questions and provide a rich description of the impact of alcohol related injuries in the four community populations. Qualitative inquiry will be used to explain significant, non-significant or surprising quantitative results. Advantages of the sequential explanatory design include its strong quantitative orientation and the link to emergent approaches where the qualitative phase can be designed as a result of the outcomes of the initial quantitative phase [13].

Study #1 will address questions 1 & 2:

1. What have been the numbers and types of injuries suffered by community people and have these changed in recent years, particularly from 2006?

2. What has been the role of alcohol in these injuries?

This study will provide the foundation data for the overall study. Following the methodology of Gladman et al. [4], who conducted a clinical audit in the 1990s, clinical files will be accessed via the community healthcare facilities in the four communities and audits will be performed for the time periods January 2006 to December 2008 and January 2009 to December 2011 to capture the pre and post timeframe of the recent alcohol controls in the communities. One of the proposed key outcomes of implementing AMPs was to reduce violence in communities, in particular violence against women and children [14,15]. A clinical file audit of this time period would capture any reduction in injury rates and provide indicators of violence rates in the communities.

The quantitative clinical file audit will search files for information related to injury generally and alcohol related injury in particular. A pre-post AMP implementation evaluation of the numbers, rates, types and outcomes of alcohol related injury will be conducted. Census data (2006 and 2011) for each community will be used to identify the study population and changes. Quantitative data management and analysis will be performed using SPSS.

Study #2 will address question 3:

3. What impacts have alcohol related injuries had on individuals and families?

The quantitative data from Study #1 will be reviewed to identify individuals with an alcohol related injury who will be invited to complete two short interviews using questionnaires that assess resilience and the impact of event. These quantitative tools will be scored as per each tool’s published protocol.

● The *Connor-Davidson Resilience Scale (CD-RISC)* is used for assessing resilience in adult populations and is comprised of 25 items rated on a 5-point scale (0–4). Higher scores indicate greater resilience [16]. The CD-RISC has been tested extensively in a number of populations and was found to have sound psychometric properties, and to be capable of distinguishing between people with greater and lesser resilience [16]. Prior testing shows good internal consistency and test-retest reliability with Cronbach’s alpha =0.89 indicating adequate consistency [16]. The CD-RISC will be used to assess individual levels of resilience.

● The *Impact of Event Scale- Revised (IES-R)* is a self-report measure of current subjective distress in response to a specific traumatic event in adults. The 22 item scale assesses three specific subscales which indicate distress and impact of event ‘Intrusion, Avoidence and Hyperarousal’ and is scored using a five point scale 0 = Not at all; 1 = A little bit; 2 = Moderately; 3 = Quite a bit; 4 = Extremely. The IES-R has high internal consistency (alpha ranging from .87 to .94) and has been translated and validated into various languages and shows good transferability across populations [17]. It will be used to access for trauma resulting from alcohol related injury.

Participants identified from Study #1 who have an alcohol related injury will be recruited by word of mouth or via a letter of invitation. If, as was found in the earlier study (4) a majority of adults had suffered injuries and that just over 50% these were alcohol related, there may be around 1850 potential participants in study #2. In this group, a sample size of 350 will permit estimates of the prevalence of high resilience and high current subjective distress with a precision of +/− 5% with adequate study power (80%), 95% confidence, allowing for the effects of the cluster sampling approach required.

Study #3 will address questions 4 & 5:

4. What is the role of resilience for individuals and families when a family member has an alcohol related injury?

5. What strategies can enhance resilience to minimise any future negative effects of alcohol related injuries?

Qualitative narrative data collection will occur through semi-structured interviews with a purposive sample of individuals and their families. A story telling or yarning approach will be used as this has been shown to be an appropriate technique with Indigenous participants [18]. From the narratives, key points pertinent to impact of injury and resilience will be coded and extracted.

The quantitative results from Study #2 will be analysed for participants who had high or low scores on the CD-RISC and the IES-R scale for inclusion into Study #3. Family members will be included in this qualitative phase to help capture the true impact of alcohol related injuries on the individual and the family and any strategies that individuals and families use to help combat the negative effects of alcohol related injuries. It is estimated that a sample size of *n* = ~30 interviews will be required before data saturation is reached. The final sample size will be calculated based on the results of Study #2 and narratives will continue until data saturation occurs.

Study #4 Delphi Study:

Community engagement, consultation and collaboration are key principles in Indigenous research [19]. The Delphi technique is an accepted means of data collection and designed to function as a group communication process which addresses a real world issue [20]. The aim is for a group of experts to achieve consensus on a topic, process, interventions or change [20]. The process of ranking or prioritising in order of importance is repeated until consensus is achieved, however the literature shows that often three iterations is sufficient to achieve consensus [21].

The Delphi study will engage community experts/leaders with a view to prioritising potential resilience strategies identified in Study #3 that could be used more widely across the community, and elsewhere, in order to improve resilience to alcohol related injury. Delphi panel members will be provided the list of strategies identified through Study #3 and asked to prioritise them. The responses will be collated into a single anonymous list or sets of lists. The collated list will be sent back to panel members with the request to reprioritise. It is envisaged that the each community will identify some similar resilience strategies however there will not be a homogenous list that fits all communities. The process will be repeated as many times as required to gain community consensus regarding resilience strategies that are applicable and could be used to improve resilience. This approach aligns with Australia’s NHMRC Road Map Action Areas I “Improving the participation of Aboriginal and Torres Strait Islander people in NHMRC programs” and II “Capacity exchange” [22].

A panel of up to 20 members per community will be approached to participate in the Delphi study including community leaders, Elders, Indigenous Healthcare Workers and other experts.

## Data analysis

Mixed method data analysis involves the analysis of both quantitative and qualitative data [13]. Each data set is analysed using the appropriate method of analysis, however the analysis is dependent on the design of the study. A sequential explanatory design involves two major sequential phases of data collection the purpose of analysing data sequentially is so that the first database informs the second database [13]. The quantitative and qualitative phases are supportive of each other and are intrinsically linked to the success of the study.

### Quantitative data

Quantitative data analysis will occur during and after the quantitative phase of the study. Data from the survey instruments will be individually scored using the scoring tool supplied [16,17]. Numerical data will be described using means, standard deviation (SD) and confidence intervals (95% CI) when approximately normally distributed and using median and inter-quartile range (IQR) when skewed. Categorical variables will be described by percentages. Standard deviations for participants and family members will be adjusted for the clustering effect of family. Statistical analysis will be conducted using PASW (SPSS version 22; IBM SPSS; Chicago, Illinois) and STATA release 13.

### Qualitative data

Thematic analysis will be used for identifying, analysing and reporting themes or patterns within data [23]. Thematic analysis is appropriate when little is known of a phenomenon [24]. The interviews will be transcribed verbatim and two researchers will individually analyse the raw data and then work together to reach a consensus on codes [25]. Standard thematic qualitative data analysis procedures will be followed where the research team will generate codes and then themes from the generated data.

## Timeline

The study will be conducted over a minimum three year period commencing with the clinical file audit and progressing through the study sequences. This time line includes reporting.

## Outcomes & Significance

Despite the implementation of alcohol reduction strategies in remote Indigenous communities, the process has been challenging and the long term successes uneven or not yet known. Although there have been fragmented attempts to look at the impact of injury related to alcohol, there has been no rigorous or systematic approach applied to date. This has led to anecdotal evidence being available which has had little or no impact on reporting or policy development. It is imperative that a well-structured research program explores the long-term impact of alcohol related injuries in these communities. The timeliness of this study is highly significant as the Queensland Government seeks an exit strategy from the Alcohol Management Plan approach. Future governments may propose different alcohol management programs for Indigenous communities making it important to capture the current situation so benchmarking can occur. This research will contribute significant understandings to the policy debates regarding future alcohol controls in Indigenous communities. For individuals, families and communities, this project gives voice to the impacts of alcohol related injuries suffered and also identifies the strengths used when individuals and families are faced with the challenges of coping with the consequences of injuries.

## Discussion

This project has the potential to address key factors in Indigenous health [26]. For Indigenous Australians it provides an understanding of the impacts of alcohol related injury and violence and highlights the benefits of positive changes for individuals, families and communities and how they can be sustained.

## Competing interests

All authors declare that they have no competing interests.

## Authors’ contributions

CW led the design and writing of the study protocol for funding, is the study manager, and coordinates data collection, data management and data analysis. CW prepared and processed the Ethics applications, and coordinated manuscript production. AC and KU participated in the conceptual process of the study and in its design and provided mentorship. All authors were involved in revising the manuscript for important intellectual content and read and approved the final manuscript.

## Pre-publication history

The pre-publication history for this paper can be accessed here:

http://www.biomedcentral.com/1471-2458/14/479/prepub
